# Health economic evaluation of a randomized controlled trial (EMBLA study), an internet-based treatment for provoked vulvodynia

**DOI:** 10.1038/s41598-023-33406-6

**Published:** 2023-04-17

**Authors:** A. Hess Engström, N. Bohm-Starke, M. Buhrman, U. Högberg, A. Skalkidou, S. Lagenskiöld

**Affiliations:** 1 Department of Women’s and Children’s Health, Uppsala University, BOX 593, 751 24 Uppsala, Sweden; 2 Centre for Clinical Research, Västmanland County Hospital, Uppsala University, Västerås, Sweden; 3 Division of Obstetrics and Gynecology, Department of Clinical Sciences, Karolinska Institute, Solna, Sweden; 4 Division Clinical Psychology, Department of Psychology, Uppsala University, Uppsala, Sweden; 5 Department of Medical Sciences, Uppsala University, Uppsala, Sweden

**Keywords:** Health care, Medical research

## Abstract

Internet-based treatment (IBT) for provoked vulvodynia (PVD) may reduce pain during intercourse and increases pain acceptance. However, a there is still a knowledge gap regarding the cost-effectiveness of IBT for PVD. The aim of this study was to perform a health economic evaluation of guided internet-based intervention for PVD as an addition to standard treatment. The sample consisted of 99 women with a PVD diagnosis. Healthcare related costs, health-related quality of life, and quality-adjusted life-years (QALYs) and incremental cost-effectiveness ratio (ICER) were analyzed. After the IBT, the intervention group had fewer visits to a midwife than the control group (*p* = 0.03), but no between-group differences were found for visits to other professionals, treatment length, health-related quality of life, QALYs, and costs for treatment. It was estimated a cost of 260.77 € for a clinical meaningful change in pain acceptance. Internet-based treatment as add-on to clinical treatment may lower number of visits to a healthcare.

## Introduction

Provoked vulvodynia is the most common cause of vulvar pain. It mainly affects women aged between 20 and 30 years, and the prevalence in Europe is 10–16%^1^. The condition is characterized by vulvar pain during physical contact for instance through intercourse or use of tampons or tightfitting clothing^2,3^. Further, provoked vulvodynia may have a negative impact on partner relationships and psychological well-being^4–6^. Its etiology remains unclear, but a combination of biological and psychosocial factors may be involved in the development and persistence of symptoms^7,8^.

The recommended treatment is a multimodal approach to provide medical management, physiotherapy, and psychosexual counselling^8^. Studies investigating different treatment such as pelvic floor physiotherapy, cognitive behavioral therapy (CBT), and mindfulness-based cognitive therapy (MBCT) found positive effects on pain reduction and sexual function^9–15^.

Internet treatments for chronic pain based on acceptance and commitment therapy (ACT), a form of cognitive behavior therapy, have shown promising results regarding functioning, pain, and psychological symptoms^16,17^. ACT focuses on openness (e.g., acceptance), awareness (e.g., mindfulness), and activity to promote behavior change and achieve psychological flexibility^18,19^. Psychological flexibility is defined as acting in accordance with personal goals and values, in the presence of potentially interfering thoughts and feelings, and with a greater appreciation of what their current situation or context allows. Thus, psychological flexibility is made up of processes related to acceptance, cognitive defusion, self-as-perspective or context, present moment awareness, values-based action, and committed action^19,20^.

Up to this point, to our knowledge, only one randomized clinical trial investigating the effects of internet-based treatment for provoked vulvodynia could be identified^21,22^. The results of that study indicated that internet-based treatment for provoked vulvodynia reduced pain during intercourse and increased pain acceptance^21,22^. This form of treatment has been described as credible and helpful to manage vulvodynia symptoms^23^. However, there is a knowledge gap regarding the cost-effectiveness of internet-based treatment for provoked vulvodynia. This is an important consideration, as provoked vulvodynia is a costly disease with high rates of comorbidities and poor quality of life^24^. Healthcare may vary in staffing levels across different geographical areas, for example regarding midwives and specialist physicians^25^. As the availability of treatment varies across regions, internet-based treatments may promote more equal treatment offers^8^. Implementing internet-based treatment for these patients could mean easy access to treatment. In the present study, it was hypothesized that participants that underwent an internet-based intervention while waiting for clinical treatment would present fewer visits to clinical treatment.

## Aim

The aim of the present study was to evaluate a guided internet-based intervention for provoked vulvodynia as an addition to usual treatment, from a health economic perspective.

## Materials and methods

### Design

This health economic evaluation has a healthcare service perspective and is based on data from the EMBLA study, a multicenter randomized study that investigated the effects of a 6-week guided internet intervention for provoked vulvodynia^21^. The study was approved by the Ethical Regional Board in Uppsala, Sweden (registration numbers 2015/031 and 2020–07,179), and registered at clinicaltrials.gov (protocol ID EMBLA, ID NCT02809612, 22/06/2016). All experiments were performed in accordance with the Declaration of Helsinki and General Data Protection Regulation (GDPR). Reporting in the manuscript followed the recommendations in the CONSORT 2010 guidelines.

### Participants

The sample consisted of 99 women with a diagnosis of provoked vulvodynia who had previously participated in the EMBLA study^22^. An informed consent was obtained from all included participants in the study. Inclusion criteria were: age over 18 years, symptoms of provoked vulvodynia for at least 6 months before enrollment in the study, access to computer and internet, and having a Swedish personal identity number. Potential participants were excluded if a diagnosis could not be confirmed through a screening interview over the telephone, if the participant was undergoing a medical investigation or treatment for provoked vulvodynia at the time of recruitment, if the participant was not fluent in the Swedish language or presented severe or acute mental illness or substance abuse. Previous treatment for provoked vulvodynia was not an exclusion criterium. Participants were randomized to either a guided internet intervention (n = 52) or standard care (n = 47) by a simple randomization process, using a computer-generated list of random numbers. Randomization was carried out until at least 26 participants were assigned to each group in each timepoint. A full description of the study protocol is published elsewhere^21^.

Participants were recruited from waiting lists from gynecological clinics in central Sweden or via social media. As previously reported, there were no differences between groups in sociodemographic data or symptoms of provoked vulvodynia at baseline, but the groups varied significantly regarding their attempts to have intercourse, where the intervention group reported fewer attempts at intercourse than controls^22^. The sample consisted of young women (mean age 24.5, SD = 4.4), most of whom were in a relationship and had a higher education. Detailed information regarding the background characteristics of the sample is given elsewhere^22^.

### Intervention

The intervention consisted of a 6-week guided internet treatment delivered during the waiting period for clinical treatment. The internet-based treatment was based on an ACT manual for treating patients with chronic pain and adapted for patients with provoked vulvodynia by an interdisciplinary team^26^. The treatment program was divided in six modules, one per week, containing both information and exercises. In every module, the participants had access to patient education that was delivered written form or throug videos. The content had explanations about the condition, pelvic floor anatomy and function, stress, chronic pain, sexuality, communication with the partner, and ACT. Every module contained different information and exercises related to ACT. They were also instructed to perform daily pelvic floor exercises focusing on relaxation, exposure exercises, and mindfulness. Exercises based on ACT were offered throughout the whole treatment. Guiding was provided through weekly written feedback from eCoaches. The participants could also contact the eCoaches by written message through the platform in case they had any questions. The intervention is described in full elsewhere^21,22^.

### Sample size calculation

The sample size calculation has been previously published elsewhere^21^. Those calculations estimated that 26 participants were needed in each group at each timepoint to identity a clinical improvement of at least 1.2 units on a visual analogue scale for vulvar pain. Calculations were based on a power of 80% for differences between groups, a significance level of *p* < 0.05, and a 20% dropout rate.

### Data collection and outcome measures

Sociodemographic data and self-reported medical history were collected via online assessments at baseline and from medical records.

To analyze healthcare utilization after the internet-based treatment, the number of visits to healthcare related to provoked vulvodynia and the total treatment length were retrieved from medical records from the gynecology clinics that referred the participants to the study. Participants recruited via social media (n = 8), and those who did not reside in the areas included in the multicenter study were invited to answer an online questionnaire with related questions regarding healthcare utilization due to provoked vulvodynia, filled out after the participants had completed the internet-based treatment. Visits to healthcare were further categorized based on the profession visited (gynecologist, midwife, physiotherapist, psychologist/counselor).

Data on quality of life and pain acceptance were collected at three timepoints: baseline (pre-treatment), post-treatment (6 weeks after baseline assessment), and follow-up (9 months after post-treatment assessment). Health-related quality of life was assessed using the questionnaire EQ5-D-3L (EuroQoL 5-dimension, 3-level), which has good construct validity and consists of five questions or domains, mobility, self-care, usual activities, pain/discomfort, and anxiety/depression, each with three possible ratings^27,28^.

To estimate costs related to provoked vulvodynia treatment, the operation managers at the gynecology clinics involved in the multicenter randomized study were invited to answer a questionnaire with questions regarding the average time spent per visit for each profession. To estimate the costs for each visit, data on the salary for each profession were retrieved from Statistics Sweden and calculated including social fee.

Costs of the intervention were estimated by adding up the costs for eCoaches, cost for consultation with senior clinicians or researchers, and costs for IT support.

Pain acceptance was assessed with the questionnaire CPAQ-R (*chronic pain acceptance questionnaire—revised*), with data collected at baseline, post-treatment, and follow-up. The questionnaire has good psychometric properties and is divided into the subscales activity engagement and pain willingness^29–32^. Activity engagement relates to performing activities despite experiencing pain, and pain willingness assesses attempts to control pain. There is no cut-off score for CPAQ-R. Higher scores indicate higher pain acceptance.

### Statistical analyses

Patient characteristics at baseline were analyzed with Mann–Whitney’s U test and the chi-square test for differences between groups.

Chi square test was used to analyze differences between groups regarding clinical treatment after the internet intervention (yes/no). Healthcare utilization due to provoked vulvodynia after internet-based treatment (number of visits) and treatment length (in months) were analyzed with Mann–Whitney’s U test as the data were not normally distributed. Analyses were carried out for the total number of healthcare visits and the number of visits by profession (gynecologist, midwife, physiotherapist, psychologist/counselor) due to a discrepancy in resources at different clinics. Due to the variation in the number of visits to midwives and physiotherapists these variables were categorized into groups (0 visits, 1–3 visits, 4–6 visits, and at least 7 visits) and then analyzed with Mann–Whitney’s U test.

Health-related quality of life assessed with EQ5-D-3L was first analyzed for differences between groups at all timepoints using Mann–Whitney’s U test without any imputation. Then, quality-adjusted life-years (QALYs) were calculated by multiplying the individual value for the quality of life index derived from EQ5-D-3L domains by the time (in years) that each participant was part of the study^33,34^. The treatment time was adjusted for participants who only completed part of the treatment. For dropouts, data were assumed and imputed as follows: 3 weeks for dropouts before post-treatment assessment and 24 weeks for dropouts between post-treatment and follow-up assessments. A dropout analysis for the randomized study has been previously reported^22^. The difference between groups regarding QALYs was calculated using Mann–Whitney’s U test.

Costs for treatment were reported in euros. To eliminate differences in price levels between countries, the Purchasing Power Parity for the years 2016–2020 and the mean purchasing power parity over the same period were used as the index for conversion from Swedish kronor to euros, covering the Euro zone as calculated by OECD 36^35^. Differences between groups in costs were analyzed with Mann–Whitney’s U test, as data distribution was skewed.

Pain acceptance (CPAQ-R) was used as a measure of clinical improvement. Results from CPAQ-R have previously been published elsewhere^22^, but are in the present study translated into a measure indicating the value to patients, i.e., calculating a reliable change index (RCI) as described by Jacobson & Truax (1991)^36,37^. An index higher than 1.96 was considered a clinically meaningful improvement. The differences between groups regarding RCI were calculated with Fisher’s test.

In the cost-effectiveness analysis, it was estimated what additional costs were needed with the treatment in comparison to waiting list for clinical treatment in order to gain one meaningful change in pain acceptance for the patients. The meaningful change in pain acceptance was estimated using RCI, as described above. The ICER was estimated using the equation:

$$ICER\; = \;\frac{{\left( {{\text{mean total costs intervention}}{-}{\text{ mean total costs control}}} \right)}}{{({\text{percentual RCI }} > { }1.96{\text{ intervention }} - {\text{ percentual RCI }} > { }1.96{\text{ control}})}}$$

Analyses were carried out using SPSS IBM Statistics, version 26.

## Results

There was a statistically significant difference between groups regarding contact with a physiotherapist before enrollment in the study, mostly due to muscle- and joint-related pain, with the intervention group reporting having contact with a physiotherapist more often than the control group (*p* = 0.02). There was a difference between groups regarding recruitment sites (U = 827, z = − 2.04, *p* = 0.04, n intervention = 49, n control = 42). More participants from site 1 and recruited via social media were randomized to the intervention group (Table 1). Sites 1, 3, and 4 had access to gynecologists, midwives, a physiotherapist and a psychologist. Site 2 did not have access to neither a psychologist nor a physiotherapist. At site 3, where most of the participants were recruited, a group meeting led by a midwife and a physiotherapist was offered before clinical treatment was initiated. This same site had a somewhat standardized number of visits to a physiotherapist, with most patients receiving three visits.

**Table 1:** Participant characteristics.

Variables[n (%)]	Total (n = 91)	Intervention (n = 49)	Control (n = 42)
Recruitment sites*			
Site 1	6 (6%)	6 (12%)	0 (0%)
Site 2	3 (3%)	1 (2%)	2 (5%)
Site 3	66 (73%)	31 (64%)	35 (83%)
Site 4	8 (9%)	4 (8%)	4 (10%)
Social media	8 (9%)	7 (14%)	1 (2%)
Contact with healthcare before enrolling in the study (self-reported)			
Psychologist	36 (40%)	19 (39%)	17 (42%)
Counselor	39 (43%)	23 (47%)	16 (39%)
Physiotherapist**	40 (44%)	27 (55%)	13 (32%)
Clinical treatment after the intervention			
Yes	71 (84.5%)	34 (79%)	37 (90%)
No	13 (15.5%)	9 (21%)	4 (10%)
Contact with gynecologist for other reasons			
Yes	18 (24%)	9 (23%)	9 (24%)
No	58 (76%)	30 (77%)	28 (76%)

*p = 0.04 for differences between groups regarding recruitment sites (Mann Whitney’s test).

**p = 0.02 for difference between groups regarding previous contact with a physiotherapist (chi-square test).

After the internet-based treatment, 79% (n = 34) of the participants in the intervention group and 90% (n = 37) of the participants in the control group received clinical treatment (*p* = 0.15).

**Figure 1:**
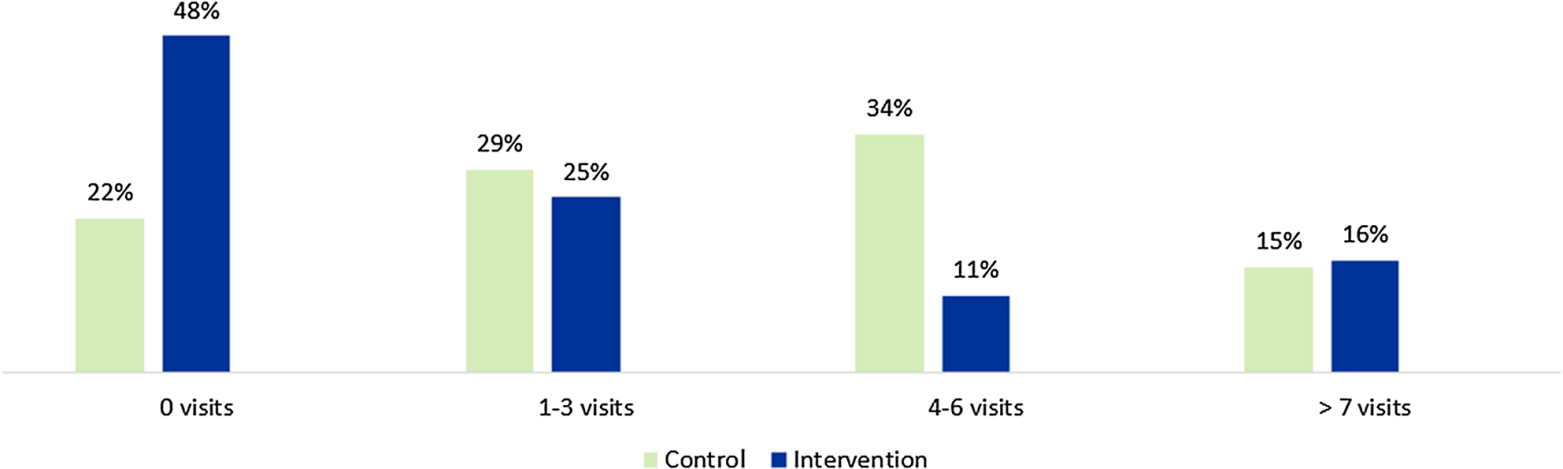
Visits to a midwife for clinical treatment.

The mean total number of visits to healthcare due to provoked vulvodynia after the internet-based treatment did not differ between groups (mean intervention group = 5.77, mean control group = 6.78) (Table 2). There was a statistically significant difference between groups regarding visits for treatment with a midwife when the data was categorized by the number of visits, where the intervention group had fewer visits than the control group (U = 665,50, z = − 2.167, *p* = 0.03, n intervention = 44, n control = 41) (Fig. 1). No differences concerning the number of visits to other professions were found.

**Table 2:** Contact with healthcare due to provoked vulvodynia.

Visits related to provoked vulvodynia after intervention	Total	Intervention	Control	*p* value
Total number of visits				
[n, median (IQR)]	5 (1.5–9)	4 (1–9)	7 (4–8.5)	0.16
N	85	44	41	
Gynecologist [n (%)]				
0 visit	68 (80%)	35 (79%)	33 (81%)	0.87
1 visit	14 (17%)	7 (16%)	7 (17%)	
2 visits	3 (3%)	2 (5%)	1 (2%)	
N	85	44	41	
Midwife [n (%)]				
0 visits	30 (35%)	21 (48%)	9 (22%)	0.03*
1–3 visits	23 (27%)	11 (25%)	12 (29%)	
4–6 visits	19 (22%)	5 (11%)	14 (34%)	
≥ 7 visits	13 (15%)	7 (16%)	6 (15%)	
N	85	44	41	
Physiotherapist [n (%)]				
0 visits	24 (28%)	16 (36%)	8 (20%)	0.27*
1–3 visits	51 (60%)	22 (50%)	2 (71%)	
4–6 visits	3 (4%)	2 (5%)	9 (2%)	
≥ 7 visits	7 (8%)	4 (9%)	1 (7%)	
N	85	44	3 41	
Psychologist/counsellor [n (%)]				
0 visits	81 (95%)	41 (93%)	40 (98%)	0.37
1–3 visits	3 (4%)	3 (7%)	0 (0%)	
4–5 visits	1 (1%)	0 (0%)	1 (2%)	
N	85	44	41	
Treatment duration (months)				
[median (IQR)]	8 (4–12.50)	7.5 (4–12.75)	9 (3.5–12.50)	0.82
N	69	32	37	
Received treatment from a physician at first visit (visit for diagnosis—medicine or advice) [n (%)]				
Yes	45 (53%)	23 (52%)	22 (54%)	Not tested
No	31 (36%)	13 (30%)	18 (44%)	
N	85	44	41	

*Mann–Whitney’s test for categorized data.

**Figure 2:**
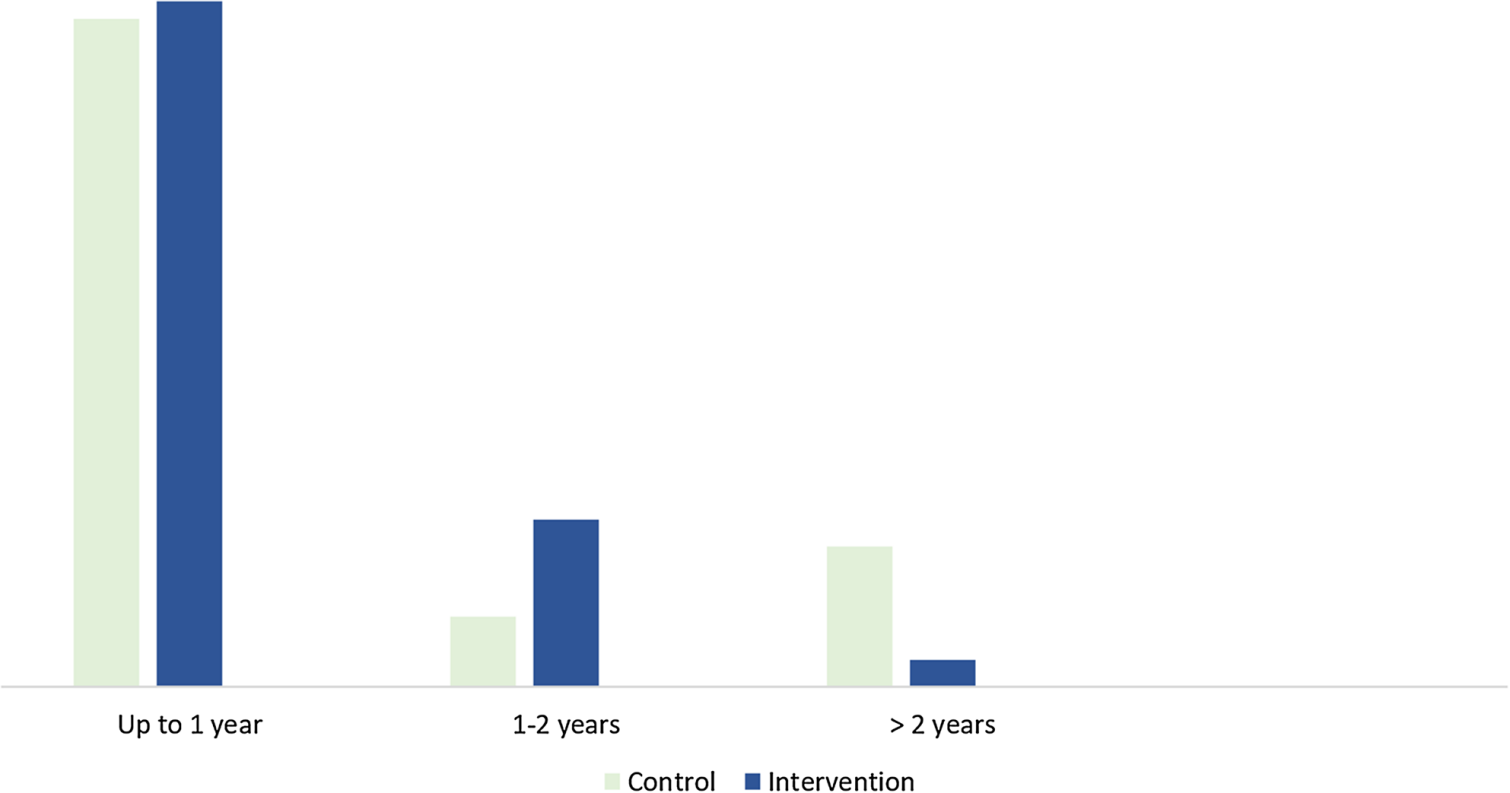
Clinical treatment length after the internet-based treatment.

The groups did not differ statistically in mean treatment length (mean intervention group: 9.47 (SD = 7.33), mean control group: 11.22 (SD = 11.01) (Fig. 2). Just over two thirds of the participants completed the clinical treatment within a year (78% in the intervention group versus 76% in the control group). In the intervention group, 19% completed the clinical treatment in 1–2 years versus 8% in the control group, while 3% had treatment for at least two years versus 16% in the control group. More than half of the participants in both groups received treatment (advice or medical treatment) from a gynecologist at their first appointment.

There was no statistically significant difference between groups regarding health-related quality of life (EQ5-D-3L) or QALYs at any timepoint (U (n intervention = 33, n control = 30) = 398.50, z = − 1.34, *p* = 0.18 at post-treatment, and U = 309, z = − 0.974, *p* = 0.33, n intervention = 27, n control = 27, at follow-up) (Table 3).

**Table 3:** Differences between groups measured with EQ5-D index and QALYs.

	Baseline		Post-treatment		Follow-up	
	Intervention	Control	Intervention	Control	Intervention	Control
EQ5-D index [mean (SD), n]	0.64 (0.25) 47	0.58 (0.32) 42	0.65 (0.21) 33	0.54 (0.33) 30	0.57 (0.30) 27	0.53 (0.35) 27
*p* value		0.84		0.29		0.57
QALYs [mean (SD), n]		–	0.71 (0.29) 33	0.59 (0.34) 30	0.75 (0.30) 27	0.65 (0.35) 27
*p* value		–		0.18		0.33

**Figure 3:**
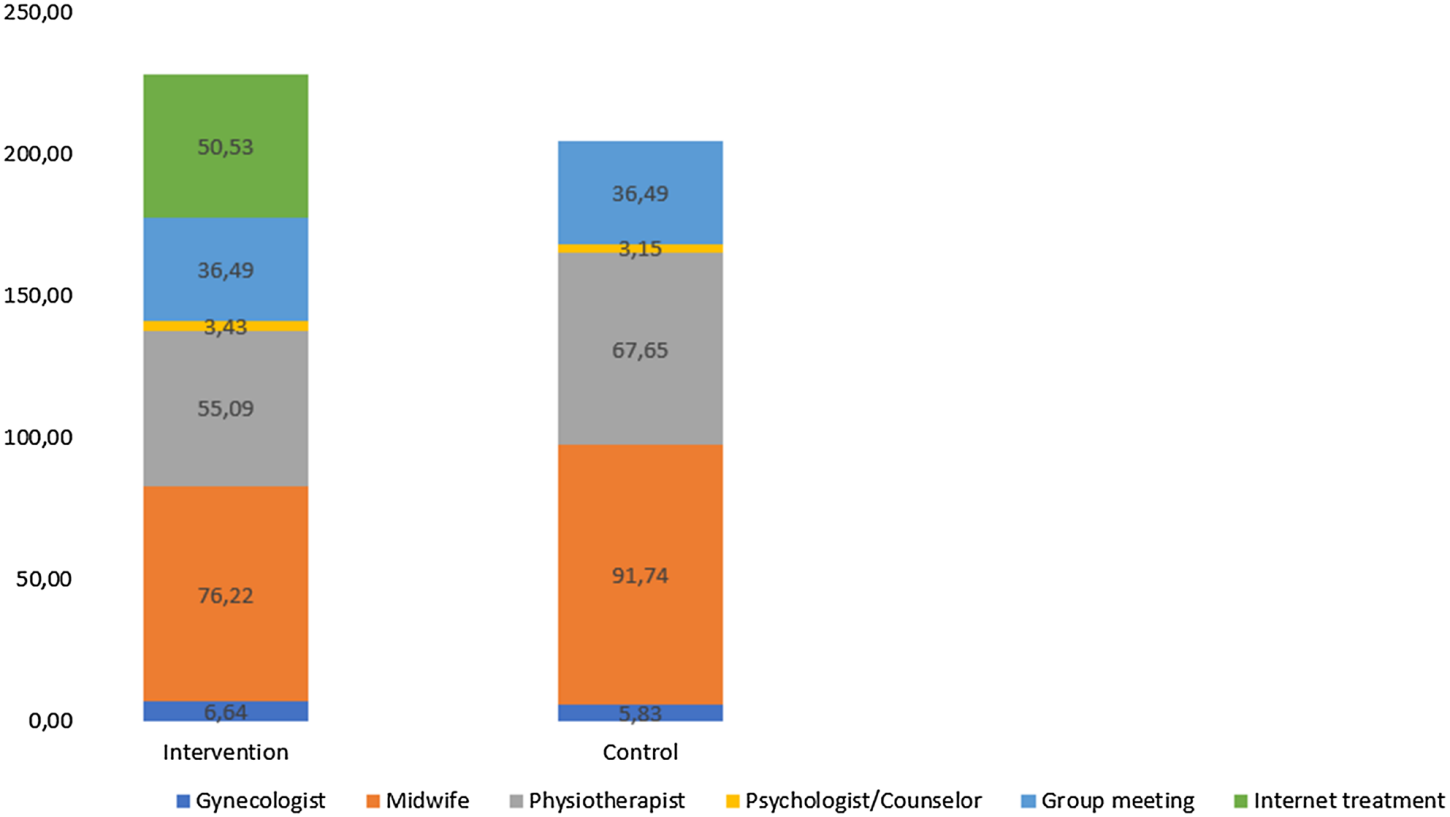
Mean costs for provoked vulvodynia treatment.

The mean difference in clinical treatment costs across groups without the internet-based treatment was 27.06 €, where the mean cost for clinical treatment for the intervention group was 154.79 € (SD = 150.65 €) and that for the control group was 181.85 € (SD = 133.83 €). When adding the costs for the internet-based treatment, the mean difference between groups in costs was 23.47 €, with the intervention group presenting the higher cost. There was no statistically significant difference between groups regarding costs related to clinical treatment even when costs for the internet-based treatment were added to costs for the clinical treatment (Tables 4 and 5, and Fig. 3).

**Table 4:** Costs for the internet intervention per patient based on salary per hour and time per patient.

Type of costs for the internet-based treatment	Costs in Euros
eCoach (time for contact with participants and salary)	34.04
Consult with a senior psychologist, gynecologist, physiotherapist (Time for contact between the eCoach and a senior colleague)	2.81
IT support	13.68
Total costs per patient	50.53

**Table 5:** Costs for clinical treatment.

Type of cost	Costs in Euros [mean (SD)]		*p* value
	Intervention (n = 43)	Control (n = 41)	
Standard care	154.79 (150.65)	181.85 (133.83)	0.17
Internet treatment and clinical treatment	205.32 (150.65)	181.85 (133.83)	0.85
Protocol-driven costs			
Visits for diagnosis and treatment	182.88 (155.78)	213.70 (136.05)	0.13
Visits for diagnosis, treatment, and internet treatment	232.79 (156.47)	213.70 (136.05)	0.55

No statistically significant differences between groups regarding RCI were found for CPAQ-R total scale or the subscales activity engagement and pain willingness at any timepoint (Table 6). At post-treatment, the intervention group presented a clinically meaningful improvement in pain acceptance (CPAQ-R total score) of 16% versus 7% in the control group (Table 6). For the subscale activity engagement, the clinically meaningful improvement was 19% in the intervention group and 4% in the control group at post-treatment. For the subscale pain willingness, 13% in the intervention group and 19% in the control group reached a clinically meaningful improvement. At follow-up, a higher percentage of participants increased their pain acceptance, but the differences between the intervention group and the control group decreased.

**Table 6:** Percentage of clinical meaningful change measured with reliable change index (RCI) for CPAQ-R.

	CPAQ-R Total score, post-treatment (n i/c = 32/27)	CPAQ-R Total score, follow-up (n i/c = 26/27)	Activity engagement, post-treatment (n i/c = 32/27)	Activity engagement, follow-up (n i/c = 26/27)	Pain willingness, post-treatment (n i/c = 32/27)	Pain willingness, follow-up (n i/c = 26/27)
Intervention	16% (5)	31% (8)	19% (6)	35% (9)	13% (4)	23% (6)
Control	7% (2)	22% (6)	4% (1)	33% (9)	19% (5)	15% (4)
*p* value	0.44	0.54	0.11	1.00	0.72	0.50

In the cost-effectiveness analysis, it was estimated that a clinical meaningful change in pain acceptance can be achieved at a cost of a cost of 260.77 € in expectancy.

## Discussion

This study evaluated an internet-based treatment for provoked vulvodynia from a health economic perspective. After the internet-based treatment, the intervention group reported fewer visits to a midwife than the control group, but no statistically significant differences between groups regarding the total number of visits to different healthcare professions, treatment length, health-related quality of life, QALYs, or costs for treatment.

In the present study, the groups differed at baseline regarding previous contact with a physiotherapist, where the intervention reported more often contact with a physiotherapist than the control group. Patients with provoked vulvodynia are a heterogenous group and care seeking behavior may vary in this population^38,39^.

Most of participants in the sample received clinical treatment after the intervention period, which is in line with results of a previous study, where participants of the internet-based treatment for vulvodynia described a need for continued contact with healthcare to work on remaining symptoms^23^.

Previous studies investigating internet interventions based on CBT for chronic pain demonstrated a reduction in symptoms and medical care use and that this form of treatment can be cost-effective when compared with face-to-face CBT^40,41^. In the present study, the mean total number of visits in the intervention group was 5.77 versus 6.78 in the control group, which was lower in comparison to another study investigating healthcare utilization due to vulvodynia (with an average of 8.8 visits per patient and year)^24^. One possible explanation for this difference may be the inclusion criteria. Only patients with provoked vulvodynia were included in our study, whereas 40% of the sample in the study by Xie et al. consisted of patients with provoked vulvodynia and 60% had generalized vulvodynia or mixed types, which may affect the need for care^24^.

The intervention group had fewer visits to a midwife than the control group, suggesting that the internet-based treatment may reduce the need for contact with healthcare, which corroborates the results of another study investigating an internet intervention based on CBT for fibromyalgia in Sweden^42^. This finding is especially relevant when taking the shortage of midwife resources in the Swedish healthcare setting into account^25^. In Sweden, midwives are a part of multidisciplinary teams working with patients with provoked vulvodynia. As specialized physiotherapists and psychologists in gynecological clinics are at short supply, midwives are used as a substitute in treating these patients. Therefore, when analyzing the number of visits and treatment length, it may be wise to take into consideration differences in available resources between different sites and how it may impact healthcare resources utilization and visits to other professions. In a previous study, after contact with a physician, treatment by a physiotherapist was the most common among patients with provoked vulvodynia, followed by treatment by a psychologist^3,24^. In our study, only a few patients had contact with a psychologist, but it cannot be determined if the reason for this was a lack of resources. In other studies, contact with psychologists for CBT has been neglected because of a scarcity of resources, despite guidelines^38^.

There was no difference between groups regarding health-related quality of life, but as the sample was small and EQ-5D-3L is not sensitive to change, this was expected^43^. Health-related quality of life was lower than in a previous study for patients with vulvodynia, but was similar to that seen in a Swedish population with fibromyalgia after an internet intervention^24,42^. Finding a health-related quality of life index for these patients may be important for future health economic evaluations of women with provoked vulvodynia.

Due to the few numbers of participants rating pain during intercourse in the randomized study, the calculation the incremental cost-effectiveness ratio was analyzed using pain acceptance as clinical measure to determine a meaningful clinical change. However, pain acceptance is an important clinical measure as it has been associated with lower pain severity and greater sexual functioning in women with vulvodynia^5^. Despite a lack of statistical significance, a meaningful clinical improvement in pain acceptance was found in 16% of the participants in the intervention group, suggesting that internet-based treatment may have the potential to improve pain acceptance at a low cost. The lack of significance in statistical tests may be explained by power issues, as the present study was not dimensioned for pain acceptance as primary outcome.

The cost-effectiveness analysis considered differences in costs in relation to clinical effects and indicated that treatment did not aggravate symptoms, but rather could improve them. Despite the lack of statistical significance between groups regarding costs for treatment, the estimated costs to achieve clinical meaningful improvements in pain acceptance was low. This result suggested that the internet-based treatment may be a valuable complement to clinical treatment, which is in agreement with the results of another study investigating internet-delivered ACT for chronic pain^44^. Further, as the costs for treatment are sunk in the second year, the cost difference may become even larger from then onward. Considering that over 20% of the participants needed more than a year to complete their clinical treatment, it can be assumed that costs for treatment and the burden on healthcare for the treatment of vulvodynia will be lower over time and follow the number of patients undergoing internet-based treatment.

### Clinical implications

Internet-based treatment for provoked vulvodynia is a low-cost intervention that requires minimal healthcare resources. As internet-based treatment can be delivered during the waiting period for clinical treatment, it does not compete with it and can instead be used as a resource for treatment optimization. The treatment’s accessibility and flexibility may help patients access evidence-based treatment from different geographical areas, which can be especially useful for patients living in areas with long waiting lists. The total costs for the intervention group were not lower in comparison to the control group. However, the difference in costs between groups were low and there is a difference in a favor of the group who received internet-based treatment regarding number of visits to healthcare as well as pain measures^22^. Further, given that the treatment for provoked vulvodynia may be long and access to specialized services might not be available in all geographical areas, the small difference in costs are justifiable taking into consideration benefits to these patients.

Internet-based treatment may, therefore, work as a complement to clinical treatment for provoked vulvodynia.

Lastly, around 50% of women with provoked vulvodynia seek help and some may spend years before receiving a diagnosis, with about half never receiving a diagnosis^45–47^. It can be hypothesized that internet-based treatment would lower the barrier to seeking care and engaging in treatment, but this should be investigated in future studies.

### Strengths and limitations

Despite this being a small study, a strength is that it follows guidelines for health economic evaluations in digital health. We translated the clinical outcome to a value for patients and then valued the resources needed to provide the treatment^48^. Other strengths of this study are the use of a randomized design and the use of a validated and relevant clinical measure in addition to the instrument for assessing general health-related quality of life. Despite the small sample size, this is a novel study, and it can give directions on how to proceed with health economic evaluations of internet interventions for provoked vulvodynia. Further, the present study may be used for sample size estimation in future studies.

A limitation of the present study concerns the differences between groups regarding baseline characteristics, distribution among different sites, and differences in available resources at the recruitment sites. It is still unclear if need of healthcare was affected by differences between groups regarding care-seeking behavior at baseline, treatment approaches at the different clinics, or the fact that participants in the intervention group were encouraged to continue practicing the exercises on their own after the intervention period.

Another limitation regards the costs for development of the internet-based treatment program and server. These costs were not included as part of the costs for treatment as we aimed to evaluate the clinical value in relation to costs, i.e., the costs for treatment if the internet-based treatment was implemented in clinical practice as a complement to usual care. Moreover, the infra-structure was already in place when the intervention was delivered as part of the regular Swedish digital healthcare system. However, infra-structures within healthcare systems may vary in different countries, as do of course other parameters, such as costs for healthcare personal. This can result in difficulties in generalizing our results to different geographical contexts, especially regarding cost-effectiveness of the intervention.

It is worth to note that the visits to the clinic may cost more than what was stated in this study as only flexible costs were considered but not premises and operating costs. If so, the clinical costs were underestimated in this study.

## Conclusion

Internet-based treatment as add-on to clinical treatment may lower number of visits to healthcare.

Larger studies investigating cost-effectiveness of internet-based treatments for provoked vulvodynia as well as meaningful clinical changes across treatments and if clinical and cost-related effects persist over time are recommended.

## Data Availability

The datasets generated during and/or analysed during the current study are available from the corresponding author on reasonable request.
